# Effect of Phenolic Compounds from *Cymbopogon citratus* (DC) Stapf. Leaves on Micellar Solubility of Cholesterol

**DOI:** 10.3390/molecules27217338

**Published:** 2022-10-28

**Authors:** Sandrine Da Ressurreição, Sónia Pedreiro, Maria Teresa Batista, Artur Figueirinha

**Affiliations:** 1 Polytechnic of Coimbra, Coimbra Agriculture School, 3045-601 Coimbra, Portugal; 2 Faculty of Pharmacy, University of Coimbra, Azinhaga de Santa Comba, 3000-548 Coimbra, Portugal; 3 Research Center for Natural Resources, Environment and Society (CERNAS), Coimbra Agriculture School, 3045-601 Coimbra, Portugal; 4 REQUIMTE/LAQV, R. D. Manuel II, Apartado, 55142 Oporto, Portugal; 5 CIEPQPF, FFUC, Pólo das Ciências da Saúde, Azinhaga de Santa Comba, 3000-548 Coimbra, Portugal

**Keywords:** spectrophotometric methodology optimization, in vitro cholesterol absorption evaluation, *Cymbopogon citratus* leaf, polyphenols activity

## Abstract

Dyslipidemias are one of the risk factors for cardiovascular diseases, the leading cause of death and hospitalization worldwide. One way to control cholesterol levels is to control the exogenous cholesterol intake in the body. Natural polyphenolic compounds, namely theaflavins from plant extracts such as black tea, showed the ability to inhibit the formation of the micellar structure, essential for the absorption of cholesterol in the intestine. There are several methodologies to determine this effect, many of which are expensive and time-consuming. Due to these facts, the main purposes of this work were to optimize an inexpensive colorimetric method to study, in vitro, the micellar solubility of cholesterol and applied it to plant extracts. In this work, *Cymbopogon citratus* leaf extracts, its phenolic fractions, and flavonoids were evaluated. The non-delipidified infusion (CcI) obtained a maximum percentage of micelle destruction of 59.22% for a concentration of 50 μg/mL and the delipidified infusion (CcdI) obtained a maximum percentage of micelle destruction of 58.01% for a concentration of 200 μg/mL. In the case of the fraction of phenolic acids (CcPAs), 23.85% of maximum micellar destruction was recorded for the concentration of 100 μg/mL, while for the fraction of flavonoids (CcF), the micellar destruction was 92.74% at 1 μg/mL, and for the tannin fraction (CcT) of 99.45% at 25 μg/mL. Luteolin presented a percentage of micelle destruction of 94.83% in the concentration of 1 ng/mL, followed by luteolin-7-*O*-glucoside with 93.71% and luteo-lin-6-*C*-glucoside with 91.26% at the concentrations of 25 ng/mL and 50 ng/mL, respectively. These results suggest the capability of polyphenols from *Cymbopogon citratus* to prevent the cholesterol absorption in the gut by micellar destruction, and its contribution for cholesterol-lowering activity.

## 1. Introduction

Cardiovascular diseases are the leading cause of death and hospitalization in developed countries, and are increasing [1]. One of the many risk factors is dyslipidemia, which is mainly associated with the formation of arteriosclerosis, plaques that settle and accumulate in the arteries. Thus, a decreasing amount of blood circulates through the arteries, with total artery blockage leading to strokes and myocardial infarction. Hypercholesterolemia is a risk factor and is often associated with the consumption of a diet rich in lipids, a lack of exercise, hypertension, diabetes, and the consumption of great amounts of alcohol, factors that have been shown to be the primary causes of heart diseases [1,2].

Cholesterol can be absorbed from foods of animal origin (i.e., fatty meats, bacon. and butter) or synthetized in the liver. On average, the cholesterol absorption rate is approximately 50%, varying widely in the human population from just 25% to about 80%. depending on the diet and genetic factors [3,4]. Most cholesterol in food is present in its esterified form. Its absorption is made through the intestinal mucosa together with the other lipids after its emulsion and incorporation into the micelles. Only non-esterified cholesterol is incorporated into micelles. Thus, a cholesterol ester hydrolase from enterocytes converts the cholesterol to the free form. After digestion by pancreatic lipase, micelles containing free cholesterol and other lipids are absorbed through enterocytes and incorporated into chylomicrons [5,6,7]. Currently, there is a great variety of medications to control hypercholesterolemia. Nonetheless, the adverse effects of these medications can often cause a weak patient acceptance and high abandonment rate, with many searching for natural products to achieve the same effect [8,9].

*Cymbopogon citratus* (DC.) Stapf., a plant native to Sri Lanka and southern India, is widely distributed in tropical climate zones of the American and Asian continents, and in temperate climate zones of the European continent. It is normally grown as an ornamental and aromatic plant, but is also used as an aromatic agent in the cosmetic industry due to its characteristic citrus odor [10,11,12]. It has a wide range of ethnomedicinal applications, namely, antispasmodic, analgesic, anti-inflammatory, antipyretic, diuretic, anxiolytic, antihypertensive and sedative, but it is not commonly used to control cholesterol [12,13,14]. An aqueous extract of fresh leaves of *Cymbopogon citratus* exhibited a hypolipidemic effect in rats, but the bioactive compounds were not identified, nor their mechanisms of action [15]. In another study, an ethanol extract of fresh leaves of *Cymbopogon citratus* demonstrated hypocholesterolemic potential in in vivo assays. The authors suggested that the modification of cholesterol absorption in the intestine, the conversion of cholesterol to bile acids, and the increased excretion of the latter were possible mechanisms of action [16]. The hypolipidemic potential of *Cymbopogon citratus* was also demonstrated in a rat model with acute hyperlipidemia, suggesting that flavonoids and tannins could be responsible for the observed effect [17]. To test the hypothesis suggested by the studies already carried out on *Cymbopogon citratus*, where the possible mechanism of action is the modification of cholesterol absorption in the intestine by its phenolic compounds, we optimized an inexpensive in vitro colorimetric method to study the micellar solubility of cholesterol in plant extracts, and applied it to the evaluation of *Cymbopogon citratus* leaf extracts, its phenolic fractions, and flavonoids on the micellar solubility of cholesterol.

## 2. Results and Discussion

### 2.1. Phytochemical Characterization of Cymbopogon citratus Phenolic Fractions

**Figure 1:**
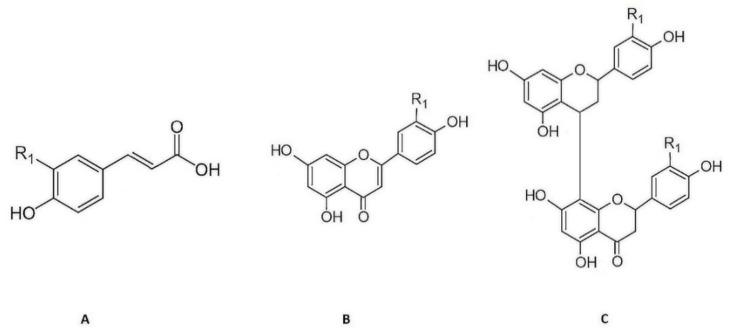
Chemical structure of the principal phenolic compounds present in aqueous extracts of *Cymbopogon citratus* leaves: (**A**) Hydroxycinnamic acid derivatives of caffeic (R_1_ = OH), ferulic (R_1_ = O-CH_3_), and *p*-coumaric (R_1_ = H) acids; (**B**) flavone derivatives of luteolin (R_1_ = OH) and apigenin (R_1_ = H); (**C**) flavan derivatives of luteoliflavans (R_1_ = OH) and apigeniflavans (R_1_ = H) (all the structures were obtained from Marvin version 21.17.0, ChemAxon (https://www.chemaxon.com, (accessed on 7 September 2022)).

The phenolic compounds present in aqueous extracts of *Cymbopogon citratus* leaf have already have been previously identified by our research group using high performance liquid chromatography coupled to photodiode-array and electrospray ionization mass spectrometry detectors (HPLC–PDA–ESI/MS^n^) [18,19,20]. The phenolic acids detected were *p*-coumaric acid, caffeic, and ferulic acid derivatives, and the flavonoids detected were of the flavone type, essentially *C*- and *O*-glycosylated derivatives of luteolin [18,19]. Finally, the tannins detected were mostly of the condensed type, namely, type B procyanidins. Derivatives of apigeniflavans and of luteoliflavans were also detected (Figure 1) [20].

**Figure 2:**
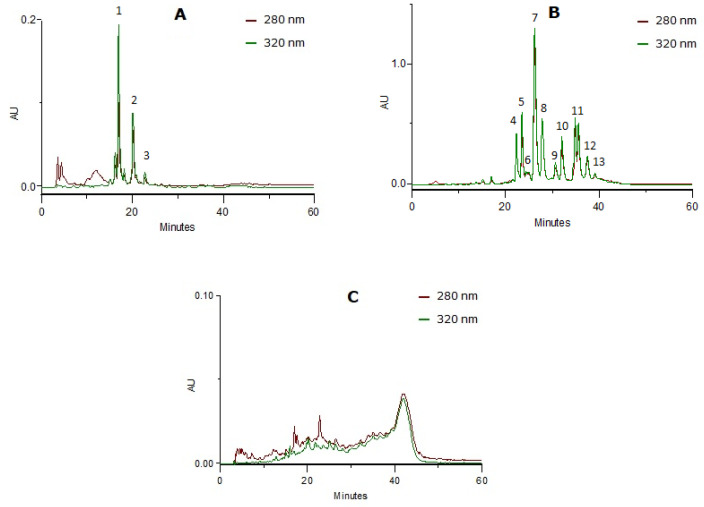
HPLC-PDA profiles at 280 nm and 320 nm from the enriched-fractions in phenolic compounds: (**A**) Phenolic acids (peaks 1 and 2: caffeic or ferulic acid derivatives; peak 3: *p*-coumaric acid derivative); (**B**) flavonoids (peaks 4, 6 to 9 and 11 to 13: luteolin derivatives; peaks 5 and 10: apigenin derivatives); (**C**) tannins.

The fractionating method of the delipidified infusion (CcdI extract) resulted in the separation of three phenolic classes (Figure 2): phenolic acids (CcPA fraction), flavonoids (CcF fraction) and tannins (CcT fraction), characterized by HPLC-PDA. The phenolic compounds were identified using the UV spectra profile and maximum absorption. The phenolic acid fraction was mainly constituted of caffeic or ferulic acid derivatives (near 250 and 324 nm and the shoulder at 298 nm) and *p*-coumaric acid derivatives (near 314 nm and the shoulder ca. 290 nm), and the flavonoid fraction by apigenin (near 270 and 330nm) and luteolin (near 270 and 350nm) derivatives. Relative to the tannin fraction, the baseline elevation and UV spectra with absorption at 280 nm and also to 330 nm revealed the presence of proanthocyanidins (near 278 nm) and heteroflavans (near 280 nm and 330–340 nm), previously described by Costa et al. [20]. The compounds identified were in accordance with the literature [18,19,20].

### 2.2. Method Development for In Vitro Cholesterol Micellar Solubility Assay

Based on the in vitro cholesterol micellar solubility assay described by Ikeda et al. [21], a fast and simple spectrophotometric method was optimized using the Liebermann–Burchard reaction. The method consists of verifying the integrity of the micellar structure of the presence of cholesterol in the samples. The micelles release cholesterol when affected by the samples. The cholesterol can be quantified by gas chromatography and by spectrophotometric methods. Because the Liebermann–Burchard reaction was used, the chosen cholesterol quantification method was spectrophotometry. The Liebermann–Burchard reaction, as described by other authors [19,22,23,24], was adapted to our samples by introducing adjustments, described in the following paragraphs.

#### 2.2.1. Wavelength Selection

**Figure 3:**
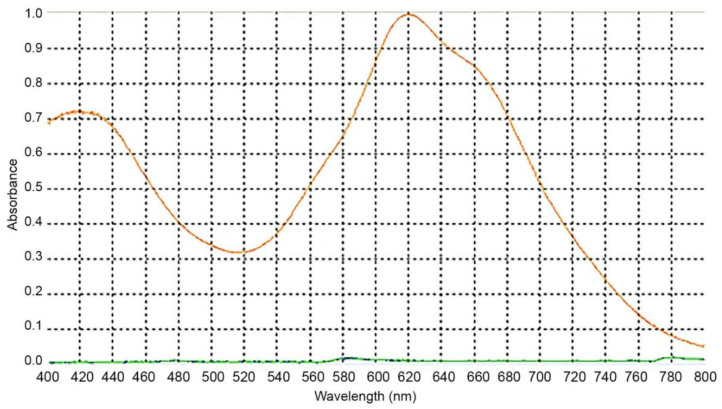
Absorption spectrum containing 750 μL of cholesterol solution with 250 μL of Liebermann–Burchard reagent.

There are several reported wavelengths in the literature for the spectrophotometric evaluation of cholesterol through Liebermann–Burchard [19,22,23,24,25]. To determine the wavelength to be used, we accessed the maximum absorbance for our assay conditions. For that, a spectral scan was performed between 400 and 800 nm (Figure 3). The 620 nm wavelength was selected to perform the readings as it corresponded to the maximum absorption, and therefore has greater sensitivity for this method.

#### 2.2.2. Cholesterol/Reagent Ratio to Be Used in the Assay

**Figure 4:**
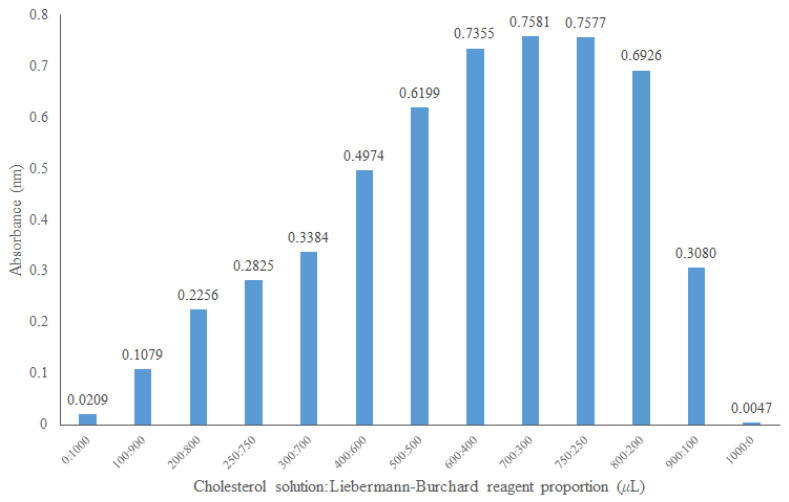
Optimization of the ratio of cholesterol at 0.5 mmol/L and the Liebermann–Burchard reagent at 620 nm.

To establish the absorbance linearity zone, the proportion of cholesterol and Liebermann–Burchard reagent was optimized. Various proportions of the 0.5 mmol/L cholesterol solution and Liebermann–Burchard reagent were tested (Figure 4). Based on this information, the proportion of 500 μL of Liebermann–Burchard reagent and 500 μL of the cholesterol solution was selected since the negative control of the micellar cholesterol solution absorbs only slightly at 620 nm according to the optimal zone of the spectrophotometer readings.

#### 2.2.3. Time and Stability of the Reaction

**Figure 5:**
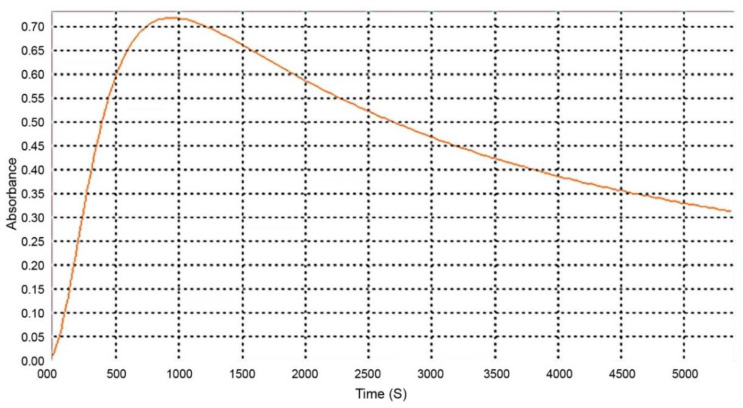
Absorption of the Liebermann–Burchard reagent with cholesterol at 620 nm over time.

Other authors have established a waiting time of 15 to 20 min after adding the reagent and before reading the absorbance [24,25,26]. To determine the stability of the Liebermann–Burchard reagent reaction with cholesterol over time, an absorbance reading at 620 nm was performed for 1 h 30 min (Figure 5). In our assay conditions, the best waiting time before reading the samples was between 15 and 16 min.

#### 2.2.4. Liebermann–Burchard Reagent Incompatibilities

Another parameter to be considered is the presence of water in the reaction medium. The Liebermann–Burchard reagent contains acetic anhydride, which in contact with water gives rise to acetic acid, inhibiting the intended reaction. Therefore, it was necessary to remove the water from our samples and resuspend them in chloroform, a solvent used by Granato and Nunes [24].

#### 2.2.5. Bile Acids Interference Evaluation

Although bile acids do not react with the Liebermann–Buchard reagent [27], 500 μL of a 6.6 mmol/L sodium taurocholate solution in chloroform was added to 500 μL of Liebermann-Burchard reagent to prevent any possible interferences or other undesired reactions. The absorbance average obtained from the three assays at 620 nm was 0.045 ± 0.001, so there was no interference of the bile acid in the experiment since this was annulled by the negative control.

### 2.3. Effect of Cymbopogon citratus Leaf Extracts and of Its Phenolic Compounds on Cholesterol Micellar Solubility

**Figure 6:**
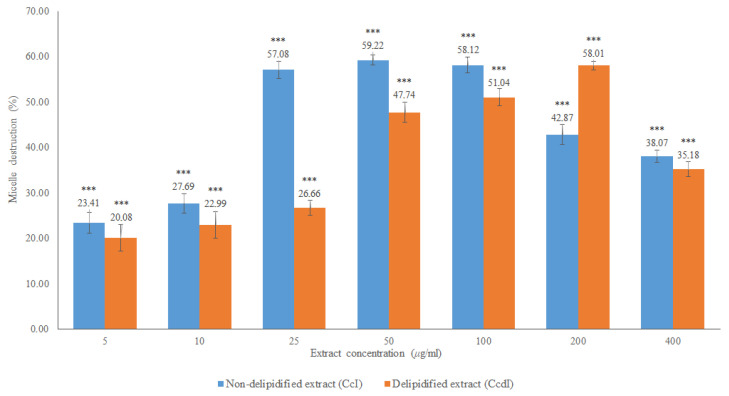
In vitro cholesterol micellar solubility assay of *Cymbopogon citratus* delipidified infusion (CcdI) and non-delipidified infusion (CcI). The results are expressed as the mean ± standard deviation (minimum of three independent assays, performed in triplicate). The control micelle destruction value was 100%. The statistical tests were performed with *** *p* < 0.05 compared to the control.

The results obtained in the in vitro cholesterol micellar solubility assay of the delipidified and non-delipidified infusions from *Cymbopogon citratus* leaf are shown in Figure 6 and Table A1.

**Figure 7:**
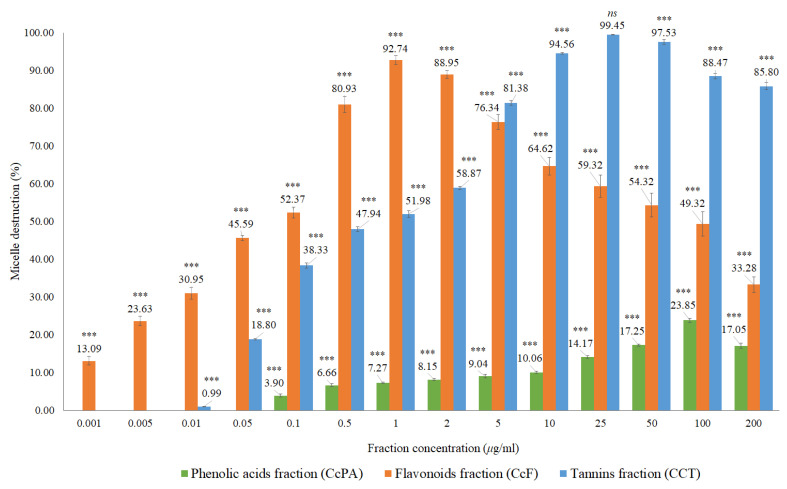
In vitro cholesterol micellar solubility assay of the CcPA, CcF and CcT phenolic fractions of *Cymbopogon citratus.* The results are expressed as the mean ± standard deviation (minimum of three independent assays, performed in triplicate). The control micelle destruction value was 100%. The statistical tests were performed with *** *p* < 0.05 compared to the control. *ns.* no significance.

Both extracts inhibited the formation of the micelles, without interference of the absorption of these extracts, which was evaluated in a control assay performed with the Liebermann–Burchard reagent. For the CcI extract, the maximum percentage of micelle destruction was 59.22% in a concentration of 50 μg/mL, while it was 58.01% for 200 μg/mL of the CcdI extract. Since *Cymbopogon citratus* leaf contains essential oil (0.5–1.13% extraction yield [28]), it is likely that there are compounds in this lipophilic fraction that also contribute to the destruction of micelles. These results reiterate the hypolipidemic effect observed in in vivo studies of *Cymbopogon citratus*. However, most of these studies do not mention which compounds are responsible for this activity or the respective mechanisms of action [15,16,29]. They also suggest the modification of cholesterol absorption in the intestine, the conversion of cholesterol to bile acids, and increased excretion of bile acids as possible mechanisms [16]. Another study demonstrated the hypolipidemic potential of *Cymbopogon citratus* in a rat model with acute hyperlipidemia and pointed out the high probability of flavonoids and tannins, being the groups responsible for the observed hypolipidemic effect, not pointing out the mechanism responsible for this action [17]. To clarify the possible contribution of *Cymbopogon citratus* phenolic compounds to the hypolipidemic effect observed in vivo as well as to suggest one of the possible mechanisms of action, the fractions of phenolic acids, flavonoids, and tannins were studied. The results obtained are shown in Figure 7 and Table A1.

Micellar inhibition was observed for all phenolic fractions, with flavonoids and tannins showing the highest percentages of micelle destruction. In the case of the fraction of phenolic acids, 23.85% of maximum micellar destruction was recorded for the concentration of 100 μg/mL, while for the fraction of flavonoids, the micellar destruction was 92.74% in the concentration of 1 μg/mL, and for the tannin fraction of 99.45% at a concentration of 25 μg/mL. Both the fractions of flavonoids and of tannins had a significant effect on micellar destruction of above 90%, which may indicate that the phytochemicals present in these fractions may contribute to the lipid-lowering activity found in the extracts of *Cymbopogon citratus*. These results corroborate that flavonoids and tannins are the main ones responsible for the hypolipidemic effect of this plant such as that suggested by Morgado et al. [17]. To our knowledge, there are no other in vitro studies to support this mechanism of action (micellar destruction) for this plant, nor for its phenolic compounds.

**Figure 8:**
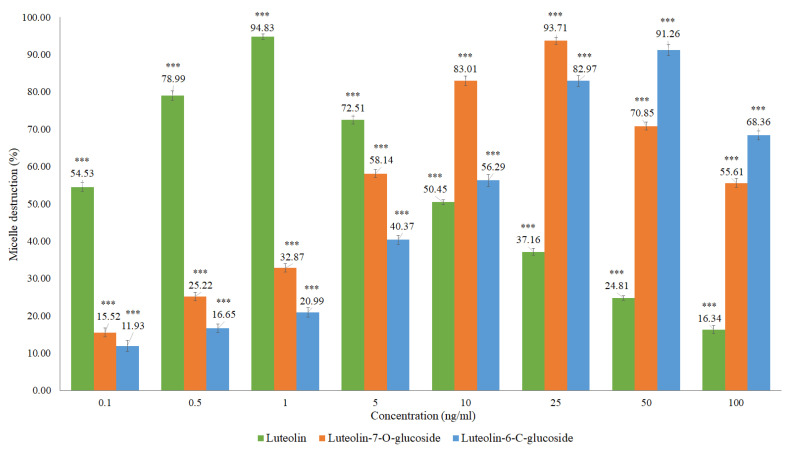
In vitro cholesterol micellar solubility assay for luteolin, luteolin-7-*O*-glucoside, and luteolin-6-*C*-glucoside (isoorientin). The results are expressed as the mean ± standard deviation (minimum of three independent assays, performed in triplicate). The control micelle destruction value was 100%. The statistical tests were performed with *** *p* < 0.05 compared to the control.

Based on the structural identification of phenolic compounds carried out in previous studies [19,20], some authentic flavonoid standards were analyzed, namely the luteolin, luteolin-6-*C*-glucoside (isoorientin), and luteolin-7-*O*-glucoside (Figure 8 and Table A1).

Micellar inhibition was observed for the flavonoids analyzed, with the luteolin showing a higher percentage of micelle destruction at a lower concentration compared to the flavonoids in glycosylated form, luteolin-6-*C*-glucoside (isoorientin) and luteolin-7-*O*-glucoside. Luteolin presented a percentage of micelle destruction of 94.83% in the concentration of 1 ng/mL, followed by luteolin-7-*O*-glucoside with 93.71% for 25 ng/mL and luteolin-6-*C*-glucoside with 91.26% in the concentration of 50 ng/mL. Flavonoids in glycosylated form are hardly absorbed in the small intestine due to their hydrophilic characteristics. These compounds are supposed to pass directly through the small intestine, being hydrolyzed by enterobacteria and releasing the corresponding genin. Non-glycosylated flavonoids can be absorbed more easily by epithelial cells of the large intestine due to their lipophilicity, which facilitates the passage through the phospholipid layer of the cell membrane [30]. The results of this research demonstrate the potential of *Cymbopogon citratus* and of the flavonoids such as luteolin and its glycosylated forms in inhibiting intestinal cholesterol absorption. Other studies have already shown that polyphenols, namely the black tea polyphenols such as theaflavin, theaflavin-3-gallate, theaflavin-3’-gallate, theaflavin-3,3’-digallate, and theaflavin-monogallates can currently be effective to decrease the plasma cholesterol concentration, inhibiting cholesterol absorption in the intestine, with at least part of the inhibition being attributed to the diminished micellar solubility of cholesterol [21]. Black tea polyphenols have cholesterol-lowering activity in humans, suppressive activity of postprandial hypertriacilglycerolemia in rats, and in vitro preventive activity in the oxidation of low-density lipoproteins (LDL) [31,32,33].

Other in vivo and in vitro studies have shown the existence of a change in the absorption of cholesterol, not by micellar destruction but by the inhibition of the epithelial cholesterol transporter Niemann–Pick C1-Like 1 (NPC1L1) by the action of flavonoids [9]. The study focused on the effect of luteolin and quercetin on cholesterol absorption mediated by the cholesterol epithelial transporter NPC1L1 in Caco-2 cells and in rats. As previously mentioned, the flavonoids present in the leaves of *Cymbopogon citratus* are *C*- and *O*-glycosylated derivatives of luteolin and glycosylated derivatives of apigenin. Epidemiological studies have shown a correlation between the consumption of polyphenols with a decreased risk of atherosclerosis due to its antioxidant effect [9]. However, the lipid-lowering activity can hardly be attributed only to the antioxidant properties of flavonoids. Nekohashi et al. [9] suggest the hypothesis that these compounds are responsible for inhibiting intestinal cholesterol transport. They started by studying the inhibition of the cholesterol absorption of 34 polyphenols in vitro in monolayer cultures of Caco-2 cells. Eleven flavonoids including liquiritigenin, sakuranetin, isosakuranetin, hesperetin, apigenin, luteolin, quercetin, daidzein, coumestrol, phloretin, and gallate of (-)-epicatechin inhibited the absorption of cholesterol in this assay, showing luteolin and quercetin to be the most active, so they were selected for further testing, namely *in vivo*. The study concluded that the luteolin and quercetin reduced high blood cholesterol levels, specifically inhibiting NPC1L1-mediated intestinal cholesterol absorption. In conventional therapy, ezetimibe is an inhibitor of NPC1L1, used for hypercholesterolemia. In this study, only genins were analyzed, namely luteolin and apigenin and not their glycosylated derivatives [9]. In another study, the authors concluded that luteolin acts on NPC1L1 through two mechanisms, namely by the inhibition of this transporter or by its expression inhibition via sterol-regulatory element-binding protein 2 (SREBP2) and hepatocyte nuclear factor 4α, leading to decreasing mRNA levels of SREBP2. Additionally, it has been reported that luteolin inhibits fatty acid synthesis from acetyl-CoA through fatty acid synthase blockage [34]. In addition, luteolin inhibits the cholesterol synthesis [31]. Catechin, a flavan-3-ol present in condensed tannins, exhibited anti-hypercholesterolemia activity through the formation of insoluble complexes and the consequent inhibition of cholesterol absorption in the gut [32]. Computational studies using density functional theory revealed that the formation of these insoluble precipitates are mainly due to charge transfer of the aromatic ring of the catechin, and by hydrogen bonds being related to the hydroxyl group number in the flavonoid [32]. Catechin, gallic acid, and epicatechin also exhibited anti-hypercholesterolemia activity through their binding to the bile acids, reducing the cholesterol solubility in micelles [33].

Our results evidenced that luteolin showed higher micellar inhibition when compared to the tested luteolin glycosides. However, among the luteolin glycosides, the *O*-glycoside showed higher activity than the *C*-glycoside. Another *O*-glycosylated flavonoid, the cyanidin-3-*O*-glucoside, also prevented the formation of micelles through precipitation [35]. When orally administered, *O*-glycosylated flavonoids are susceptible to hydrolysis in the stomach by the gastric juice as well as by intestinal enzymes, releasing the respective genin, achieving a greater activity relative to the *C*-glycosides [36,37]. However, in order to understand the underlying mechanisms of the tested luteolin glycosides, more studies are needed. Concerning tannins, the leaves of *Cymbopogon citratus* are predominantly of the condensed type. There is evidence that condensed tannins in the diet interfere with the different events involved in intraluminal lipid processing including enzymatic hydrolysis, the formation of micelles and the uptake of lipid digestion products in the intestine. However, most studies have focused on the interaction between proanthocyanidins and pancreatic lipase in vitro and, in some cases, in animal models. Therefore, further studies are needed to elucidate the role of condensed tannins in digestion and intestinal fat absorption in humans [38,39]. Knowing these compounds have the ability to complex and precipitate with different types of molecules, namely proteins and metals [40], it is possible that they may have the same type of behavior with steroids, establishing, for example, hydrogen bridges, inhibiting their absorption, which may explain the lipid-lowering action. Most ingested condensed tannins are not absorbed in the small intestine and accumulate in the colon, where they are degraded by intestinal microorganisms into low molecular weight aromatic acids, which differ according to their hydroxylation profile and the length of their aliphatic side chain. These microbial metabolites are absorbed in the colon and can also be conjugated by colonocytes or in the liver, resulting in derivatives of glucuronide, glycine, sulfate, and methylated derivatives [38,39].

## 3. Materials and Methods

### 3.1. Plant Material

Dry leaves of *Cymbopogon citratus* (DC.) Stapf. were provided by the company Ervital (Mézio, Portugal). The plant was grown in a greenhouse in the Mezio region (Castro Daire, Portugal) and was harvested in September 2013. A specimen was deposited in the herbarium of the Faculty of Pharmacy, University of Coimbra, with the reference A. Figueirinha 0109.

### 3.2. Reagents and Materials

Methanol Hipersolv^®^, absolute ethanol, and pure acetone (Merck^®^, Darmstadt, Germany) were used for the delipidified infusion fractionation. For the monitorization and characterization of fractions, methanol Lichrosolv and methanol Hipersolv^®^ (VWR, PA, USA), glacial acetic acid, ethyl acetate, formic acid (98–100%), acetone and toluene (Merck^®^), deionized water Milli-Q (Millipore Symplicity^®^, Molsheim, France), diphenylboryloxyethylamine and polyethylene glycol 4000 (Sigma^®^ Chemical Co., St. Louis, MO, USA), *p*-dimethylaminocynamaldehyde and sulfuric acid (Merck^®^) were used.

For in vitro evaluation of micellar solubility, sodium taurocholate (European Pharmacopoeia Reference Standard^®^, Strasbourg, France), phosphatidylcholine (Sigma^®^ Chemical Co., St. Louis, MO, USA), cholesterol (Sigma^®^ Chemical Co., St. Louis, MO, USA), sodium chloride (Merck^®^), sodium phosphate (Merck^®^), and chloroform (HiPersolv Chromanorm^®^, VWR, Radnor, PA, USA) were used. The Liebermann–Buchard reagent was prepared by mixing 0.5 mL of sulfuric acid (J. T. Baker^®^, Deventer, The Netherlands) in 10 mL of acetic anhydride (Panreac^®^, Barcelona, Spain), as described by [24]. Authentic standards of the flavonoids luteolin (Extrasynthese^®^, Genay, France), luteolin-6-*C*-glucoside (isoorientin) (Extrasynthese^®^), and luteolin-7-*O*-glucoside (Extrasynthese^®^) were also used.

### 3.3. Preparation of Extracts and Fractions

Dry leaves of *Cymbopogon citratus* were ground with the aid of a mill (Braun^®^ KSM2, Kronberg, Germany) and later sieved with a sieve of 250 µm (Endecotts^®^ 60 Mesh, London, UK).

An infusion was prepared by adding boiling water to the sieved material (5 g leaves/150 mL water) and letting it stand for 15 min. Then, the extract was filtered using filter paper (Whatman^®^ #1, Kent, UK). An infusion aliquot was delipidified by shaking and decanting three times with *n*-hexane (150 mL). The extracts, infusion (*Cymbopogon citratus* infusion—CcI), and delipidified infusion (*Cymbopogon citratus* delipidified infusion—CcdI) were concentrated using a rotary evaporator (Rotavapor R-114, Büchi^®^, Flawil, Switzerland) coupled to a vacuum pump (Vacuum Pump V-700, Büchi^®^) and a compact refrigeration circulator (Minichiller, Peter Huber Kältemaschinenbau AG, Offenburg, Germany). The extracts were lyophilized using a freeze-dryer (FTS Systems type EZ-DRY, USA) and subsequently stored at −22 °C, protected from light and moisture.

**Figure 9:**
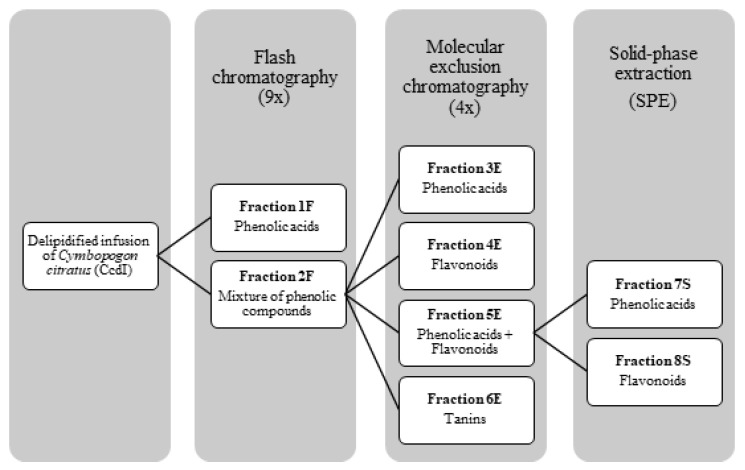
Fractionation process of phenolic compounds present in the delipidified infusion (CcdI) of the *Cymbopogon citratus* leaf.

For the CcdI fractionation, Flash column chromatography followed by molecular exclusion chromatography and solid phase extraction (SPE) were initially used. All fractionation processes were monitored by thin layer chromatography (TLC) and high pressure liquid chromatography coupled to a photodiode array detector (HPLC-PDA) for polyphenols. In this way, the CcdI extract was solubilized in 5% aqueous methanol, in the proportion of 1 g in 20 mL of solvent, and 7 mL of this solution was injected in a C18 phase reverse column (Buchi^®^, 40 × 150 mm, particles diameter of 40–63 μm) of a Flash chromatography equipment with two pumps (Buchi^®^, Pump module C-605) coupled to a UV detector (Buchi^®^, C-640), the fractionation being monitored at the wavelengths of 260, 300, and 350 nm. The mobile phases used were methanol LiChrosolv and MiliQ-water with a variable gradient of 5 to 100% of methanol. These data were acquired with ECOMAC^®^ version 0.238 (Chrastany u Prahy, Czech Republic) software. This procedure was repeated eight times, resulting in five fractions: a fraction of phenolic acids (1F) and four other fractions containing a mixture of compounds (2F) that were subsequently mixed. To separate the phenolic compounds from the 2F fraction, this was subjected to molecular exclusion chromatography using a column of 923 cm^3^ that was 5 cm in diameter and 47 cm in height, with a stationary phase of Sephadex LH-20 (235.65g) balanced with absolute ethanol. The fractionation of 2F was monitored using the wavelength of 366 nm with a UV lamp (CAMAG^®^, no. 022.9120, Muttenz, Switzerland) using absolute ethanol followed by 70% aqueous acetone as the mobile phases. Thirty-one fractions were obtained. Phenolic acids and flavonoids were eluted separately with absolute ethanol and tannins with 70% acetone, with four fractions resulting from this fractionation process according to their chemical class: phenolic acids (3E), flavonoids (4E), a mixture of phenolic acids and flavonoids (5E), and tannins (6E). The 5E fraction was subsequently subjected to SPE using a C_18_ Chromabond^®^ (10g/70mL) (Machery-Nagel^®^, Düren, Germany). Stationary phase was activated with 35 mL of methanol and 35 mL of MiliQ-water. A total of 8 mL of the 5E fraction was injected in the column using MiliQ-water, followed by 5% aqueous methanol and later 100% methanol as mobile phases. This fractionation was repeated twice. From the fractions collected, two of them contained phenolic compounds: phenolic acids (7S) and flavonoids (8S). At the end of the fractionation process, fractions containing the same type of phenolic compounds were combined, resulting in three final fractions: fraction of phenolic acids (CcPA) (obtained by joining the fractions 1F, 3E and 7S), the fraction of flavonoids (CcF) (obtained by joining the fractions 4E and 8S), and the fraction of tannins (CcT) (6E) (Figure 9). The obtained yields of phenolic acids, flavonoids, and tannins were 12.09%, 1.9%, and 2.98%, respectively.

### 3.4. Phytochemical Analysis

Extracts and fractions were monitored by TLC and HPLC-PDA, as described below.

#### 3.4.1. Thin Layer Chromatography (TLC)

The fractionating process was monitored by TLC using two systems: System S1 for flavonoids and phenolic acids [41], and System S2 for tannins [42].

##### System S1

Flavonoids and phenolic acids were monitored using pre-coated plates of silica gel with fluorescence indicator 60 F_254_ (Merck^®^, Darmstadt, Germany) and the mobile phase ethyl acetate–formic acid–glacial acetic acid–water (100:11:11:26 *v*/*v*), as described by [43]. The detection was performed with Natural Product Reagent (1% methanol diphenylboryloxyethylamine solution) (NP) and 5% ethanol polyethylene glycol 4000 (PEG) and observed under UV light at a wavelength of 366 nm. Phenolic acids were detected by a blue color. Relative to flavonoids, apigenin derivatives presented a yellow/greenish color and luteolin derivatives an orange color [41].

##### System S2

The presence of tannins was monitored using a pre-coated plate of silica gel with fluorescence indicator 60 F_254_ (Merck^®^). The plate was eluted with toluene–acetone–acetic acid (3:7.5:1) [42], and detection was performed using a 1% (*w*/*v*) solution of DMACA (*p*-dimethylaminocynamaldehyde) in methanol sulfuric acid (8 mL sulfuric acid in 100 mL methanol). Subsequently, the plate was heated at 100 °C for 5 min and then observed under visible light. In this system, the presence of tannins is verified by purple spots, and can be identified as low molecular weight tannins and high molecular weight tannins due to its retention factor (R_f_), being the highest R_f_ for high molecular weight tannins [42].

#### 3.4.2. High Pressure Liquid Chromatography Coupled to Photodiode Array Detector (HPLC-PDA)

The phytochemical characterization was performed in a Gilson apparatus equipped with a photodiode-array detector (PDA) (Gilson Electronics SA^®^, Villiers Le Bel, France) using a Spherisorb^®^ S5 ODS-2 column (250 × 4.6 mm i.d., 5 µm) (Waters Corporation^®^, MA, USA) and a Nucleosil guard cartridge C_18_ (30 × 4 mm i.d., 5 µm) (Macherey-Nagel) at 24 °C. Data treatment was performed with the Unipoint software, version 2.10 (Gilson, WI, USA). For the mobile phase, a gradient of methanol and HPLC water–formic acid (95:5) was used: methanol 5–15% (0–10 min), methanol 15–30% (10–15 min), methanol 30–35% (15–25 min), methanol 35–50% (25–35 min), and methanol 50–80% (35–40 min), followed by an isocratic elution of methanol during 20 min at a flow rate of 1 mL/min. The volume of the sample injected was 100 µL. The UV–Visible spectra were acquired between 200 and 600 nm and the chromatographic profiles recorded at the wavelengths 280 and 320 nm.

### 3.5. In Vitro Evaluation of Micellar Solubility

After optimization, the method for the evaluation of micellar solubility was applied to the CcdI and CcI extracts of *Cymbopogon citratus*, CcPA, CcF, CcT phenolic fractions, and to the standard luteolin and two glycosylated flavones, luteolin-6-*C*-glucoside (isoorientin) and luteolin-7-*O*-glucoside identified in the *Cymbopogon citratus* extracts [19].

The CcdI and CcI extracts were dissolved in distilled water in concentrations of 5, 10, 25, 50, 100, 200, and 400 µg/mL. For the CcPA fraction, concentrations of 0.1, 0.5, 1.0, 2.0, 5.0, 10.0, 25.0, 50.0, 100, and 200 µg/mL were used, while for the CcT fraction, the concentrations used were 0.01, 0.05, 0.10, 0.50, 1.0, 2.0, 5.0, 10.0, 25.0, 50.0, 100, and 200 µg/mL, and for the CcF fraction, the concentrations used were 0.001, 0.005, 0.01, 0.05, 0.1; 0.5, 1.0, 2.0, 5.0, 10.0, 25.0, 50.0, 100, and 200 µg/mL. The flavonoid standard, namely luteolin (Extrasynthese^®^, Genay, France) and two glycosylated flavones, luteolin-6-*C*-glucoside (isoorientin) (Extrasynthese^®^) and luteolin-7-*O*-glucoside (Extrasynthese^®^), were tested at the concentrations of 0.10, 0.50, 1.0, 2.0, 5.0, 10.0, 25.0, 50.0, and 100 ng/mL. 

First, a micellar solution was prepared containing 6.6 mmol/L of sodium taurocholate (European Pharmacopoeia Reference Standard^®^, Strasbourg, France), 0.6 mmol/L of phosphatidicoline (Sigma^®^ Chemical Co., St. Louis, MO, USA), 0.5 mmol/L cholesterol Sigma^®^ Chemical Co., St. Louis, MO, USA), 132 mmol/L sodium chloride (Merck^®^), and 15 mmol/L sodium phosphate (Merck^®^) at pH 6.8. After the dissolution in an ultrasonic bath (Bandelin Sonorex^®^ Tipox, Berlin, Germany), the solution was placed in an oven (Memmert^®^, Schwabach, Germany), protected from light, at 37 °C for 24 h. The samples (100 µL) were added to 3 mL of micellar solution, placed in the oven, protected from light, at 37 °C for 1 h and filtered through a hydrophilic polyvinylidene fluoride (PVDF) membrane filter with 0.22 μm (Filter-Lab^®^, Barcelona, Spain) porosity to retain the micelles [21]. Two control assays were performed: a positive control containing unfiltered micellar solution to assess the maximum absorbance possible for a sample with a total destruction of the micelles, and a negative control with filtered micellar solution to eliminate the possible interference of the micellar solution reagents and to monitor the micelle’s formation and filtration process. The samples were concentrated using a multiple sample evaporation system (Multivapor P-12, Büchi^®^) coupled to a vacuum system (Vacuum Pump V-700, Büchi^®^), and to a cooling system (Minichiller, Peter Huber Kältemaschinenbau AG^®^, Offenburg, Germany). To ensure complete removal of the water, the samples were subsequently placed in an oven (Memmert^®^) at 37 °C under vacuum and resuspended in chloroform (HiPersolv Chromanorm^®^, VWR, Wayne, PA, USA) until the initial volume was obtained. Liebermann–Burchard’s reagent was prepared in a dark flask with the aid of an ice bath by mixing 0.5 mL of sulfuric acid (J. T. Baker^®^) in 10 mL of acetic anhydride (Panreac^®^, Barcelona, Spain). To assess the cholesterol, 500 µL of sample was added to 500 µL of Liebermann–Burchard reagent, and left for 15 min protected from light for color development. Finally, the absorbance at 620 nm was read on a spectrophotometer (Cintra 101, GBC Scientific Equipment^®^, Hampshire, IL, USA; Software: Cintra 2.2 build 226, GBC Scientific Equipment^®^, Hampshire, IL, USA). The procedure was the same for the controls. The tests were carried out in three independent assays and in triplicate to each independent assay. Spectral scanning was performed between 400 and 800 nm to eliminate possible color interference in the test and that of the possible phytosterols. The results were expressed as a percentage of micelle destruction according to the following expression: Percentage of micelle destruction (%) = ((Absorbance of the sample − Absorbance of the negative control)/(Absorbance of the positive control − Absorbance of the negative control)) × 100.

### 3.6. Statistical Analysis

The results obtained were expressed as the mean ± standard deviation. For this, the Microsoft Office Excel^®^ software 16.0. developed by Microsoft Corporation^®^ (Albuquerque, NM, USA) was used to create the graphics. The statistical analysis was performed in thee GraphPad Prism program (version 5.02, GraphPad Software, San Diego, CA, USA). To evaluate the effect of micelle destruction, we performed one-way ANOVA followed by Dunnett’s test. The limit of significance was set at *** *p* < 0.05.

## 4. Conclusions

Our study suggests that *Cymbopogon citratus* extracts and their polyphenols have the capability to prevent cholesterol absorption in the gut by micellar destruction. *Cymbopogon citratus* phenolic compounds are promising for future use as an alternative to conventional treatments such as statins, which are the first line of treatment for hypercholesterolemia, but have many unwanted side effects such as fatigue, muscle pain, and gastrointestinal problems, among others. However, the mechanism of action that supports our results is not as effective as therapies acting at the level of the synthesis of endogenous cholesterol, responsible for the greatest contribution to the serum cholesterol content. Therefore, the effect is expected to be limited. Consequently, it will be more likely to be used in combination with other medicines. In fact, ezetimibe, a selective inhibitor of intestinal cholesterol absorption, is sometimes used in combination with statins when cholesterol levels are not adequately controlled with statins alone. However, therapies combining the use of this plant associated with drug therapies require additional studies and in vivo and clinical assays.

## Data Availability

Data is contained within the article or Appendix A.

## Sample Availability

Samples of the compounds are available from the authors.

## Appendix A

Table A1 in Appendix A shows a summary of the results of the in vitro cholesterol micellar solubility assay of the *Cymbopogon citratus* infusions, phenolic fractions, and flavonoid standards.

**Table A1:** In vitro cholesterol micellar solubility assay of *Cymbopogon citratus* infusions, phenolic fractions and flavonoid standards. The tests were carried out in three independent assays and in triplicate for each independent assay. The results are expressed as mean ± standard deviation.

Micelle Destruction (%)								
Concentration (µg/mL)	Non-Delipidified Extract (CcI)	Delipidified Extract (CcdI)	Phenolic Acids Fraction (CcPA)	Flavonoids Fraction (CcF)	Tannins Fraction (CcT)	Luteolin	Luteolin-7-*O*- glucoside	Luteolin-6-*C*- glucoside
0.0001	-	-	-	-	-	54.53 ± 1.21	15.52 ± 1.21	11.93 ± 1.45
0.0005	-	-	-	-	-	78.99 ± 1.26	25.22 ± 1.11	16.65 ± 1.15
0.001	-	-	-	13.09 ± 1.11	-	94.83 ± 0.74	32.87 ± 1.15	20.99 ± 1.26
0.005	-	-	-	23.63 ± 1.21	-	72.51 ± 1.07	58.14 ± 1.07	40.37 ± 1.21
0.01	-	-	-	30.95 ± 1.56	0.99 ± 0.02	50.45 ± 0.72	83.01 ± 1.21	56.29 ± 1.56
0.025	-	-	-	-	-	37.16 ± 0.89	93.71 ± 0.93	82.97 ± 1.45
0.05	-	-	-	45.59 ± 0.71	18.80 ± 0.21	24.81 ± 0.63	70.85 ± 1.07	91.26 ± 1.54
0.1	-	-	3.90 ± 0.43	52.37 ± 1.45	38.33 ± 0.65	16.34 ± 1.11	55.61 ± 1.21	68.36 ± 1.21
0.5	-	-	6.66 ± 0.41	80.93 ± 2.12	47.94 ± 0.61	-	-	-
1	-	-	7.27 ± 0.19	92.74 ± 1.15	51.98 ± 0.89	-	-	-
2	-	-	8.15 ± 0.28	88.95 ± 1.07	58.87 ± 0.38	-	-	-
5	23.41 ± 2.27	20.08 ± 2.88	9.04 ± 0.45	76.34 ± 1.95	81.38 ± 0.67	-	-	-
10	27.69 ± 2.12	22.99 ± 2.94	10.06 ± 0.29	64.62 ± 2.38	94.56 ± 0.23	-	-	-
25	57.08 ± 1.89	26.66 ± 1.66	14.17 ± 0.37	59.32 ± 2.96	99.45 ± 0.06	-	-	-
50	59.22 ± 1.15	47.74 ± 2.14	17.25 ± 0.28	54.32 ± 3.15	97.53 ± 0.63	-	-	-
100	58.12 ± 1.69	51.04 ± 1.84	23.85 ± 0.54	49.32 ± 3.25	88.47 ± 0.72	-	-	-
200	42.87 ± 2.25	58.01 ± 0.96	17.05 ± 0.72	33.28 ± 2.08	85.80 ± 0.93	-	-	-
400	38.07 ± 1.30	35.18 ± 1.68	-	-	-	-	-	-
